# A Nationwide Survey to Investigate Burnout and Quality of Life Among Thoracic Surgery Residents in Italy

**DOI:** 10.3390/healthcare13090962

**Published:** 2025-04-22

**Authors:** Giovanni Mattioni, Federico Raveglia, Andrea Onofri, Andrea Anastasi, Graziana Carleo, Diletta Mongiello, Doroty Sampietro, Cinzia Scala, Luigi Paladini, Giuseppe Cardillo, Franca Melfi, Mohsen Ibrahim, Carmelina Cristina Zirafa, Riccardo Orlandi, on behalf of the SIET Residents’ Committee Collaborative Group

**Affiliations:** 1 School of Thoracic Surgery, University of Milan, 20122 Milan, Italy; riccardo.orlandi@unimi.it; 2 Department of Thoracic Surgery, IRCCS San Gerardo Hospital, 20900 Monza, Italy; federico.raveglia@icloud.com; 3 Department of Agricultural, Food and Environmental Sciences, University of Perugia, 06121 Perugia, Italy; andrea.onofri@unipg.it; 4 Department of Mental Health, Fondazione IRCCS Ca’ Granda Ospedale Maggiore Policlinico, 20122 Milan, Italy; andrea.anastasi@unimi.it; 5 Thoracic Surgery Unit, Department of Precision and Regenerative Medicine and Ionian Area, University of Bari Aldo Moro, 70010 Bari, Italy; g.carleo2@studenti.uniba.it (G.C.); diletta.mongiello@gmail.com (D.M.); doroty.sampietro@gmail.com (D.S.); 6 Department of Thoracic Surgery, IRCCS San Raffaele Scientific Institute, Università Vita-Salute San Raffaele, 20132 Milan, Italy; scala.cinzia@hsr.it; 7 Department of Medical and Surgical Sciences, Institute of Respiratory Diseases University Hospital, 71100 Foggia, Italy; luigi.paladini@gmail.com; 8 Unit of Thoracic Surgery, Azienda Ospedaliera San Camillo Forlanini, 00152 Rome, Italy; gcardillo@gmail.com; 9 Minimally Invasive and Robotic Thoracic Surgery, Robotic Multispecialty Center of Surgery, University Hospital of Pisa, 56124 Pisa, Italy; franca.melfi@gmail.com (F.M.); c.zirafa@gmail.com (C.C.Z.); 10 Division of Thoracic Surgery, Department of Medical and Surgical Sciences and Translational Medicine, Sant’Andrea Hospital, Sapienza University of Rome, 00189 Rome, Italy; mohsen.ibrahim@gmail.com

**Keywords:** burnout, thoracic surgery, residents, training, quality of life, Italy

## Abstract

**Background**: Surgical residents are a high-risk population for burnout, yet no studies have assessed its prevalence among thoracic surgery residents in Europe or Italy. **Methods**: A nationwide cross-sectional survey was conducted among Italian thoracic surgery residents to assess burnout and quality of life. The Maslach Burnout Inventory measured burnout risk, while tailored questions evaluated quality of life. Univariate and multivariable analyses identified burnout risk factors, and χ^2^ tests explored relevant associations between variables. **Results**: Of 193 eligible residents, 98 (50.8%) completed the survey. High burnout risk was identified in 60.2% of respondents. Independent risk factor associations between burnout risk and low perceived inclusion and aggregation, low colleague quality, low residency program rating, low personal life satisfaction, perceived lack of valorization, and exposure to sexual harassment were not significant in multivariable models. No differences in burnout risk were found across gender, geographic location, or training year. **Conclusions**: Burnout among Italian thoracic surgery residents underscores systemic challenges such as excessive administrative demands, insufficient mentorship, limitations to self-care, and gaps in theoretical training. Addressing these issues requires comprehensive reforms, including curriculum enhancement, strengthened mentorship, improved administrative support, and accessible mental health resources. A multi-level intervention strategy is essential to enhance resident well-being and training quality.

## 1. Introduction

The pursuit of a career in thoracic surgery is widely acknowledged as a demanding endeavor. Numerous factors contribute to this challenge, including an intensive workload, significant responsibilities, a complex working environment, and the inherently high-stress nature of the job [1]. Eventually, these stressors can lead to burnout—an occupational syndrome particularly prevalent among healthcare professionals—which is associated with a broad spectrum of physical and mental health complications. Surgical residents are notably susceptible to burnout, and thoracic surgery residents are no exception [2]. They often face unwritten norms that emphasize self-sacrifice and an unceasing commitment to excellence for the benefit of patients and the surgical team [3]. Furthermore, the work environment may impose high demands while providing limited resources [4]. These professional challenges intersect with personal, relational, familial, and financial obligations within the life of a resident. Nonetheless, burnout among surgical residents remains a frequently underestimated issue.

Burnout manifests through a range of symptoms, including emotional exhaustion, characterized by fatigue, depletion of energy, and debilitation; depersonalization, leading to inappropriate attitudes towards patients, irritability, loss of idealism, and social withdrawal; and a diminished sense of personal accomplishment, reflected in reduced productivity, low morale, and an inability to cope effectively [5]. In healthcare workers, burnout has been associated with increased medical errors, poorer patient outcomes, reduced professionalism, job dissatisfaction, intentions to leave the profession, and higher attrition rates [6,7,8,9,10]. Additionally, burnout has been correlated with a heightened risk of substance abuse and suicide [11].

The prevalence of burnout within the general population of surgical residents is estimated to range from 38% to 69% [12,13]. Regarding thoracic surgery specifically, data are scarce, though one North American study reported a prevalence of 43% [3]. To date, no studies have examined the prevalence of burnout among thoracic surgery residents in Europe or Italy. Evidence concerning other Italian residency programs suggest an overall burnout prevalence of 67% [14], with differences across specialties such as general surgery (58%) [15], urology (49%) [16], and gynecology (61%) [17].

A nationwide Residents’ Board was established in Italy in 2023, as part of an initiative of the Italian Society of Thoracic Endoscopy (SIET—Società Italiana di Endoscopia Toracica). The aim was to investigate a wide range of residency topics (e.g., burnout, training, simulation, and quality of life). Given the absence of European and Italian evidence, a nationwide survey was conducted to collect up-to-date data. Here, we present the results of the survey related to the topic of burnout and quality of life.

To better contextualize the findings of the present study, a brief overview of the thoracic surgery residency program in Italy is provided in Appendix A.

## 2. Materials and Methods

### 2.1. Survey

A cross-sectional descriptive study was conducted through an anonymous, voluntary online survey developed with SurveyMonkey (SurveyMonkey Inc., San Mateo, CA, USA, www.surveymonkey.com) and distributed in December 2023. The survey (Supplementary Material S1) included 74 questions addressing multiple aspects of thoracic surgery residency. The survey, conducted in Italian, was sent by email to thoracic surgery residents in Italy who were in their 2nd to 5th years of residency. First-year residents were excluded to avoid confounding variables associated with proximity to the beginning of their residency (November 2023). Responses were collected until March 2024, with periodic reminders sent during this timeframe.

In accordance with local regulations, Institutional Review Board (IRB) approval was waived due to this study’s lack of patient involvement and absence of interventions on the resident cohort.

An estimate of the number of thoracic surgery residents in Italy was obtained through consultation of the online platform of the Ministry of University, which is dedicated to each annual test and provides basic data on residents assigned to the different residency programs and any reported withdrawals. These data were further confirmed through cross-checks with residents enrolled in the different residency programs who responded to our contact attempt.

### 2.2. Burnout and Quality of Life Assessment

Burnout risk was assessed in a dedicated section of the survey, validated according to the Maslach Burnout Inventory (MBI) Scale. This scale, comprising 22 items, assesses three dimensions of burnout: emotional exhaustion (EE), depersonalization (DP), and personal accomplishment (PA). Threshold values for each risk category were established based on normative MBI data for Italian healthcare professionals (low EE ≤ 14, intermediate EE = 15–23, high EE ≥ 24; low DP ≤ 3, intermediate DP = 4–8, high DP ≥ 9; low PA ≥ 37, intermediate PA = 30–36, high PA ≤ 29) [18]. A high burnout risk was defined by a high score in at least one of the two subscales of EE and DP, as described in previous studies [19,20]. A high score in both EE and DP, coupled with a low score in PA, indicated severe burnout risk. Quality of life was assessed using nine questions addressing personal life, self-care, and interactions within the working environment. These items were not derived from previously validated instruments available in the literature, but were specifically developed to explore particular aspects of the residency experience. The full set of questions is available in Supplementary Material S1 (under the heading *Quality of Life During Residency*).

### 2.3. Statistical Analysis

Frequencies were calculated for nominal data (e.g., gender), while means and standard deviations were reported for quantitative data (e.g., total working hours). Median and interquartile range (IQR) were used for Likert-scale data.

Firstly, a univariate analysis was performed. Generalized linear models with binomial error and logit link were fitted to the number of residents at risk of burnout, while potential risk factors were included as predictors (one variable per each fitting process). Likert-scale predictors were grouped into four categories: very insufficient 1–3, insufficient 4–5, sufficient/good 6–7, and excellent 8–10. The back-transformed proportion of individuals at risk of burnout with delta standard errors was calculated from the fitted model for each level of the nominal predictors.

Then, predictors with significant effects were included in a multivariable model developed via stepwise forward selection based on the Akaike Information Criterion (AIC).

Finally, potential associations between predictors were evaluated using χ^2^ tests for independence. Statistical significance (i.e., rejection of null hypothesis) was defined with a *p*-value < 0.05.

## 3. Results

**Figure 1:**
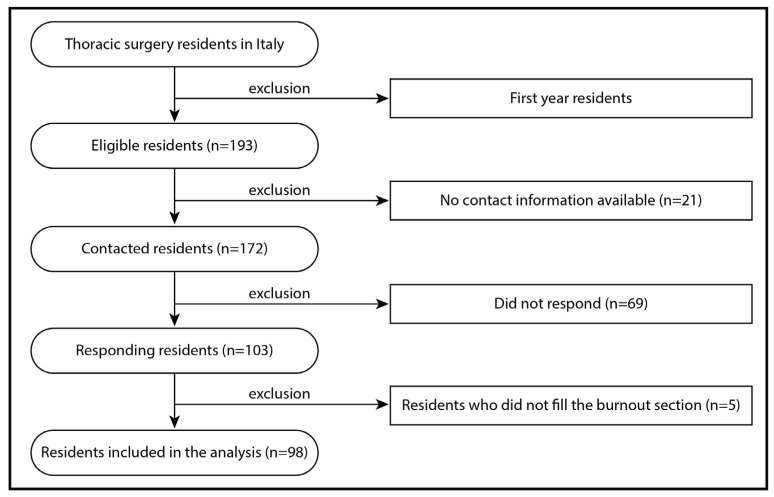
Flowchart of residents selection and participation. The survey was conducted between January and March 2024.

**Figure 2:**
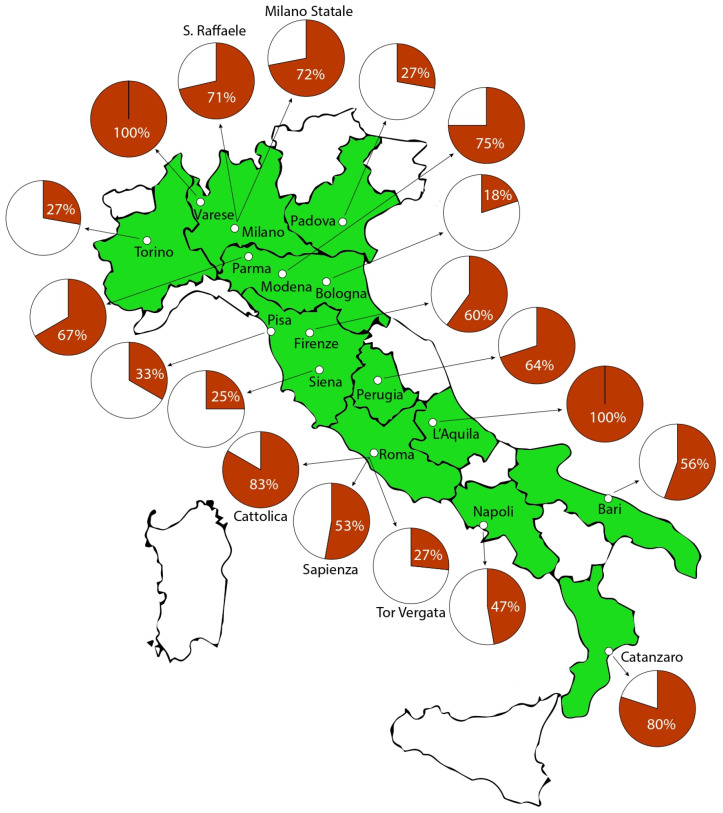
Graphical representation of residents’ participation to the survey per residency program in Italy (n° of responding residents/n° of residents). White regions do not have Universities offering a thoracic surgery residency program.

The estimated population of thoracic surgery residents in Italy in 2024, from the second to fifth year of residency, comprises 193 individuals, as detailed in Supplementary Table S1. Of these, 98 residents (50.8%) completed the survey, including the sections addressing burnout, and were thus included in the analysis. The selection of participants is detailed in the flowchart of Figure 1. The sample comprised 52 females (53.1%), with a mean age of 26.8 ± 2.6 years (median 26; range 23–41 years). The most common relationship status was a stable partnership without marriage (45.9%), and 4.1% of respondents had children, all of whom were male. Table 1 provides detailed demographic characteristics of the respondents, and their geographic distribution is illustrated in Figure 2. The list of the survey’s main questions and the related answers are available in Table 2.

### 3.1. Burnout

The median scores for burnout subscales were 24.5 (IQR 16.0–31.8) for EE, 38 (IQR 33.3–41.0) for DP, and 5.5 (IQR 2.3–10.0) for PA. Overall, 59 residents (60.2%) exhibited a high risk of burnout. Burnout was positive in one subscale in 33 cases (33.7%), in two subscales in 20 cases (20.4%), and severe burnout risk (all three subscales) was observed in six residents (6.1%). EE reached burnout levels in 51 cases (52.1%), DP in 34 cases (34.7%), and PA in 15 cases (15.3%). Of those with the lowest PA scores, only two residents (13.3%) did not exhibit burnout, while six residents (40%) in this group displayed high levels of burnout. More details are available in Table 3.

### 3.2. Quality of Life

Residents rated their overall personal life satisfaction with a mean score of 5.9 ± 1.9 out of 10 (median 6). In most cases (78.6%), residency workload limited the opportunity to engage in activities outside of work. The rating of the perceived level of aggregation and inclusion by the residency program (e.g., event organization, fostering resident interaction) received an average score of 5.9 ± 2.7 out of 10 (median 6), while the rating of the perceived valorization by the residency program scored an average of 5.8 ± 2.4 (median 6). When asked to rate the quality of colleagues (encompassing both residents and senior surgeons), respondents reported a mean score of 6.9 ± 1.6 out of 10 (median 7).

Dropout contemplation was reported as frequent or very frequent by 16% of respondents, with an additional 25.5% indicating occasional thoughts of leaving. The remaining 58% of residents reported never (31.6%) or rarely (26.5%) contemplating leaving the residency program. If given the choice again, 17.3% of respondents indicated they would not choose thoracic surgery, while 13.6% stated they would choose thoracic surgery, but in a different country. Conversely, 47.6% would choose thoracic surgery again at their current institution.

Residency significantly limited self-care and attendance to health-related appointments for 32% of respondents (“very frequently” or “frequently”), whereas 24.3% reported moderate restrictions (“sometimes”). Among respondents, 8% reported having experienced sexual harassment, with females comprising 7 of these 8 cases. Senior surgeons were the source in 50% of cases, patients in 12.5%, other residents in 12.5%, and nurses in 25%. Nearly half (48%) of respondents had at least occasionally heard comments implying that thoracic surgery was unsuitable for women or homosexual individuals.

See also Table 2 for further details.

### 3.3. Working Load

The reported mean monthly working hours were 219.4 ± 45.3 (median 220). Respondents reported dedicating a mean of 85.4 ± 62.5 h (median 80) to administrative tasks (e.g., national and international societies database compilation, organizing patient appointments, and managing patient documentation). Sixty-two percent of respondents reported to perform on-call night shifts (62%) and on-duty night shifts (22%).

### 3.4. Factors Related to Burnout Risk

**Figure 3:**
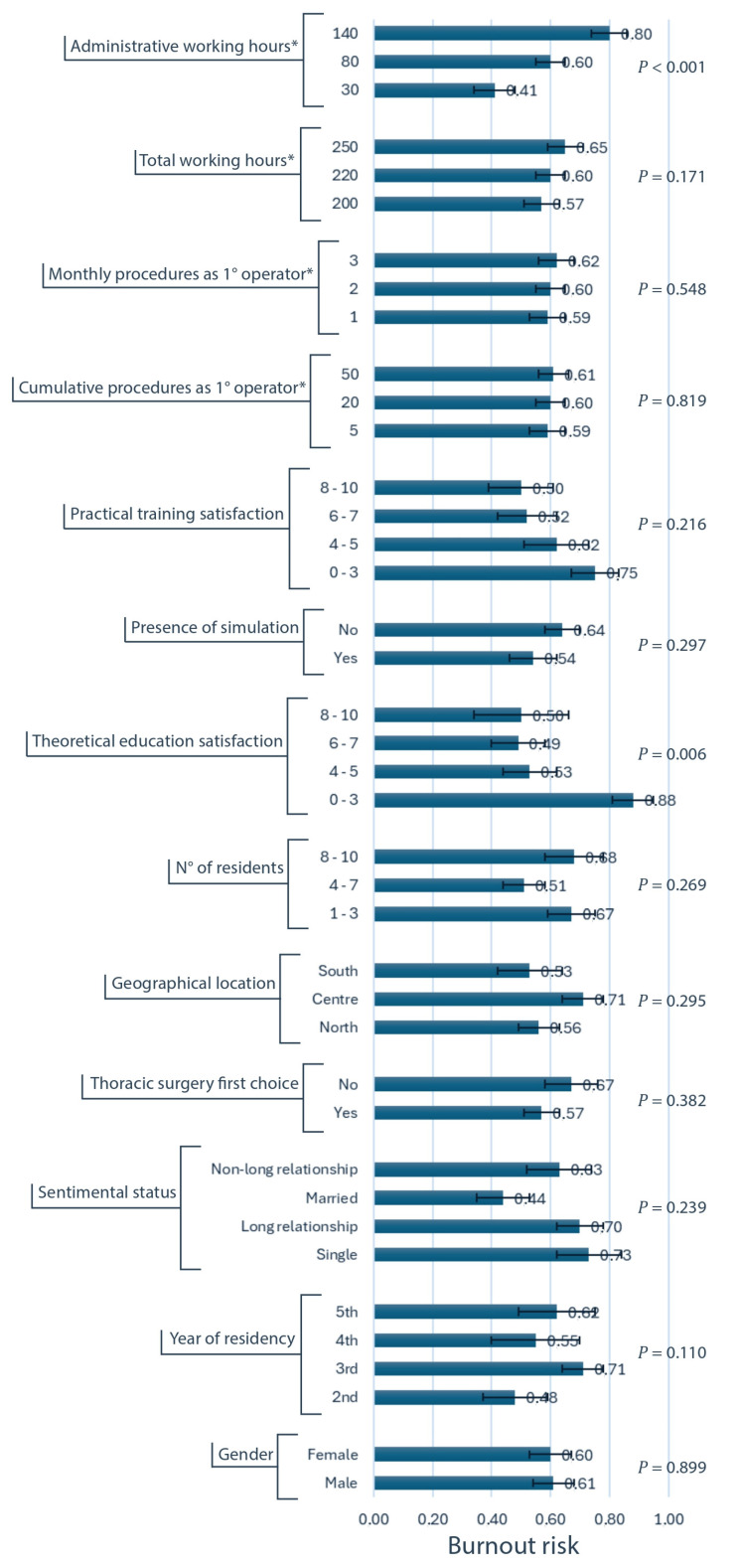
Univariate analysis of variables associated to burnout risk, with standard errors (first part). Burnout risk is represented on the horizontal axis. The exact corresponding values are reported at the right extremity of the horizontal blue columns, as well as the error bars representing standard errors. * These variables are quantitative, and the 25th, 50th, and 75th percentiles were used as categories for the analysis.

**Figure 4:**
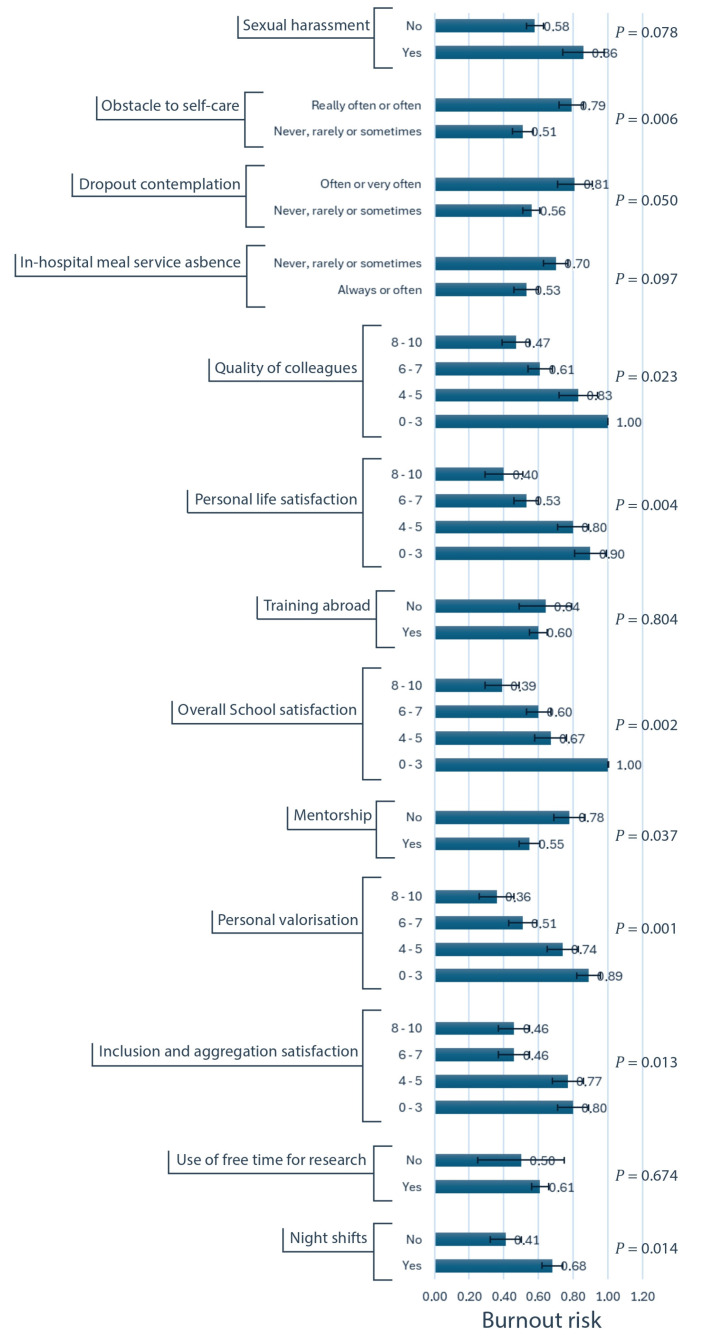
Univariate analysis of variables associated to burnout risk, with standard errors (second part). Burnout risk is represented on the horizontal axis. The exact corresponding values are reported at the right extremity of the horizontal blue columns, as well as the error bars representing standard errors.

Univariate analysis (Figure 3 and Figure 4 and Supplementary Table S2) identified several factors significantly associated with an increased risk of burnout. Increased administrative workload (*p* < 0.001) and on-call or on-duty night shifts (*p* = 0.014) were associated with higher burnout risk. Lower satisfaction ratings regarding the theoretical education offer (*p* = 0.006), the inclusivity and aggregation promoted by the residency program (*p* = 0.012), the quality of colleagues (*p* = 0.023), the residency program overall (*p* = 0.002), and personal life (*p* = 0.004) were all associated with an elevated burnout risk. Additionally, the absence of mentorship (*p* = 0.037), the perceived lack valorization from the residency program (*p* = 0.001), the absence of an in-hospital meal service (*p* = 0.097), the frequent thoughts of leaving residency (*p* = 0.049), the limitations to self-care due to workload (*p* = 0.006), and exposure to sexual harassment (*p* = 0.078) were all significantly associated with increased burnout. Notably, gender (*p* = 0.899), geographic location (*p* = 0.295), and surgical exposure (*p* = 0.551) did not demonstrate an association with burnout risk.

When incorporating all significant variables into a stepwise multivariable model (Table 4), the most influential factors associated with burnout were identified as (in order of importance) the administrative workload (Likelihood Ratio = 10.62; *p* < 0.001), the difficulty in attending to personal health needs due to workload (LR = 4.05; *p* = 0.044), the presence of on-call and on-duty night shifts (LR = 5.49; *p* = 0.019), the lack of mentorship (LR = 4.75; *p* = 0.029), and the lower satisfaction with the theoretical education provided (LR = 7.40; *p* = 0.060).

### 3.5. Associations Among Risk Factors

Several variables demonstrated statistical significance in the univariate analysis but were not confirmed by the multivariable model. This is usually related to a certain degree of association between predictors. To better understand this behavior, the χ^2^ test was used (Table 5). A higher perception of valorization was associated with a higher theoretical education rating (χ^2^ = 60.7; *p* < 0.001); while an inverse relationship was observed with access to self-care (χ^2^ = 18.4; *p* < 0.001). Personal life satisfaction and overall satisfaction for the residency program were significantly and positively correlated with theoretical education rating (χ^2^ = 23.2; *p* = 0.006 and χ^2^ = 52.5; *p* < 0.001, respectively). Additionally, having a mentor was associated with a higher personal life satisfaction (χ^2^ = 10.1; *p* = 0.018). Furthermore, having more enjoyable colleagues was linked to a higher theoretical education rating (χ^2^ = 20.6; *p* = 0.015) and better access to self-care (χ^2^ = 6.7; *p* = 0.082), while more frequent consideration of dropping out was associated with the absence of a mentor (χ^2^ = 4.8; *p* = 0.029). A higher satisfaction for the aggregation and the inclusion promoted by the residency program was associated with higher theoretical education ratings (χ^2^ = 32.1; *p* < 0.001), the absence of night shifts (χ^2^ = 14.0; *p* = 0.003), and less difficulty in self-care due to workload (χ^2^ = 11.4; *p* = 0.01).

## 4. Discussion

When performing a search on the PubMed research engine using the combination of keywords “burnout” AND “residen*” AND “thoracic”, only 28 results are displayed, of which 5 are specifically related to burnout in thoracic or cardiothoracic surgery residents. This shows the lack of evidence concerning our specific specialty. To the best of our knowledge, this study is the first to investigate burnout and quality of life among thoracic surgery residents in both Italy and Europe. Our findings show that a substantial proportion (60%) of Italian thoracic surgery residents are at high risk for burnout. This prevalence exceeds the burnout rates reported in a North American study (47%) [3], but are consistent with the literature regarding the general Italian residents’ population [14]. This burden accurately reflects the complexity inherent to thoracic surgery training. Notably, gender, the geographic location of the Residency program, and the level of surgical exposure did not demonstrate significant associations with burnout risk. We initially hypothesized that surgical exposure might influence burnout in opposing directions—either due to insufficient surgical opportunities, which could lead to frustration, or excessive exposure, which could reduce personal free time. However, neither hypothesis was confirmed by our findings.

An increased administrative workload has been consistently linked to burnout in previous studies [21,22], and this relationship was confirmed in our cohort too, in both univariate and multivariable analyses. These findings highlight the impact of non-clinical responsibilities on mental health, emphasizing the importance of balancing clinical and administrative tasks to mitigate burnout. Enhancing the administrative support in the thoracic surgery units may reduce the workload that residents are sustaining. In contrast, total working hours showed no significant relationship with burnout risk. Nevertheless, it is worth mentioning that the median workload for residents exceeds the contractual training requirements by approximately 60 h [23]; this additional work is neither compensated nor officially acknowledged. Taken together with findings related to surgical exposure, these results suggest that the risk of burnout is not necessarily linked to the volume of work but rather to the nature and perceived value of the tasks performed.

Burnout has also been associated with reduced attention to personal health and missed medical appointments among surgical residents [3,24]. In our study, approximately one-third of respondents stated that their workload significantly limited their ability to engage in self-care; these limitations were independently associated with an increased burnout risk. In comparison to North American data on cardiothoracic surgery residents [3], these results are moderately encouraging, suggesting that Italian residents may have better access to self-care despite workload demands. Nevertheless, residency programs should safeguard residents’ health, monitoring if they are prioritizing personal health care.

The relationship between night shifts and burnout, whilst predictable, remains inconsistently supported by evidence. Studies concerning senior physicians indicate that performing night shifts is associated with elevated burnout risk [25]. Although specific data on cardiothoracic surgery residents are lacking, in a study of head and neck surgery residents, frequent night shifts correlated with higher burnout risk [26]. In our analysis, the presence of on-call and on-duty night shifts was independently related to burnout risk. Our study examined the presence/absence of night shifts rather than their frequency, due to the peculiar aspect of residency in Italy, acknowledging that night shifts are neither paid nor mandatory for residents. In addition, we did not assess whether residents did benefit from post-shift rest, but it is well documented that surgical residents often remain on duty following night shifts, a practice associated with higher burnout rates [27]. Future steps should be taken to overcome the absence of retribution for residents’ night shifts, in order to increase professional responsibility and at the same time provide compensation in exchange for the work performed.

Lack of mentorship is another established factor contributing to burnout among residents [21,28], and our findings support this association. In addition, mentorship was significantly associated with a higher appreciation for the residency program and a less frequent consideration of dropping out, explaining why these latter two did not appear in the multivariable model. Notably, the likelihood of having a mentor decreased as the number of residents in a department increased; a finding from a parallel analysis from this survey focused on surgical exposure and workload. The overcrowding of thoracic surgery residency programs in Italy, which started in 2021, has inevitably led to a decline in both the quality and quantity of training. Meanwhile, the expansion of the thoracic surgery training network has not kept pace, creating a mismatch between the number of residents and the available training opportunities. As a result, mentorship, a cornerstone of surgical training, has been adversely affected, leading to lower engagement and higher risk of burnout for residents. The Italian thoracic surgery community must address this imbalance to ensure that training and mentorship remain of high quality. Potential solutions may include better planning for residents’ access to residency programs and rotations in thoracic surgery units, as well as recognizing and rewarding senior surgeons that actively promote mentorship.

Satisfaction with the surgical residency program is inversely related to burnout risk [21,29]. In our study, the quality of theoretical education was the only factor independently associated with burnout risk. Surprisingly, the rating of practical training had no statistically significant relationship, although showing a trend. Overall satisfaction was significant only at univariate analysis. However, both personal life satisfaction and overall satisfaction with the residency program were positively correlated with the theoretical education rating, explaining their absence in the multivariable model. Concerning theoretical education, residency programs should revise their curricula, incorporating more structured and accessible lectures and seminars focused on relevant and cohesive topics in thoracic surgery. Theoretical education should provide the basics to understand clinical and surgical practice, which can then be deepened through hands-on experience during routine activities. It is worth mentioning the positive direction of the recent initiative proposed by the residency program of Padua (SISCT—Scuole Italiane Specialità in Chirurgia Toracica), which set up a regular national streaming channel for thoracic surgery residents with the aim of ensuring an equal theoretical education across the whole of Italy.

Other noteworthy findings include the inability to assess the relationship between burnout risk and residents’ perception of their work–life balance, due to an exceedingly high number of respondents reporting an insufficient balance. In addition, sexual harassment was shown to still be an existing blight on residents, which was experienced by 13.5% of female respondents. In half of the instances, harassment came from senior surgeons, whereas in one quarter, from nurses. However, the literature data concerning all surgical residencies show a prevalence ranging from 10 to 55%, higher than that resulting from our cohort [12,30].

From the present analysis, a profile of the thoracic surgery resident at higher risk of burnout can be outlined. Warning factors include a high administrative workload, participation in night shifts, inadequate self-care, lack of mentorship, low personal, professional, and educational satisfaction, limited aggregation and inclusion within the residency program, low recognition, low appreciation for colleagues, frequent contemplation of dropout, and exposure to sexual harassment.

Addressing and preventing these issues requires an urgent, multi-faceted approach. Primarily, this should involve enhancing mental health support for residents (e.g., free psychological counselling, screening programs with the occupational health service, and promoting work–life balance). Additionally, all previously suggested improvements should be considered, including curriculum revisions, the implementation of an effective mentorship program, increased administrative support, and activities that promote aggregation, networking, and improved interpersonal relationships among colleagues.

It should be emphasized that any potential solution must involve every single member of the process, from the residents to the Italian Ministry. Moreover, problems should not be viewed as an individual responsibility, as this mindset will not lead us toward solving the issue. While individual factors may be involved, they should not be the sole focus of the proposed solutions [8].

On the other hand, it should be highlighted that no gender or geographical disparities in burnout risk were found. Even the year of residency showed no association, which may suggest that residents are treated equally throughout their training. Furthermore, a significant relationship with burnout risk was demonstrated only for administrative workload, but not for total working hours or higher surgical exposure. This finding suggests that residents demonstrate a high level of resilience and adaptability to the demanding work schedule of thoracic surgery residency, and that the main issue is not the quantity of work, but rather the quality of the tasks. Finally, another confirming finding is that personal accomplishment scores were notably high, which may reflect the dedication and resilience of residents in pursuing this demanding specialty, even in the face of considerable burnout risk and reduced quality of life.

Although a major strength of this survey is the high participation rate (approximately 50% of the estimated total), spread across the country, ensuring a representative sample, we are also aware of the limitations. A selection bias might have occurred given the proportion of residents who did not participate. In particular, more motivated or more dissatisfied residents could have been more inclined to respond. Unfortunately, due to the lack of access to data for all residents, a comparison between characteristics of respondents and non-respondents was not possible. Another limitation could be the recall bias, because data were self-reported and dependent on participants’ memory recall. Furthermore, most variables investigated were subjective parameters, which may have been influenced by personal motivations and contingent settings. The survey was aimed solely at residents, and therefore, all considerations are based on their perspective, which may not reflect the true reality of residency programs in Italy. We would also like to point out that while these results are the first in Europe, it is likely that they cannot be applied to all countries, given the existing differences to Italy. As a consequence, future studies in Europe are needed to further understand these findings.

## 5. Conclusions

This study highlights the significant prevalence of burnout among thoracic surgery residents in Italy, underscoring the intense pressures and demanding workload inherent to this specialty. Independent risk factors for burnout risk were excessive administrative duties, restricted access to self-care, lack of mentorship, presence of night shifts, and dissatisfaction with theoretical education. Importantly, burnout rates impact not only on residents’ mental health but also on their professional performance and commitment to thoracic surgery. To address these issues, a comprehensive approach is required: enhancing mentorship, improving both theoretical and practical training, and implementing systemic changes to balance clinical, educational, and administrative responsibilities; these are vital steps toward creating sustainable and rewarding surgical careers. Prioritizing mental well-being within residency programs is essential to create a healthier, more supportive training environment and to cultivate rewarding, sustainable careers in thoracic surgery.

## Figures and Tables

**Table 1:** Demographic characteristics of survey’s respondents.

Demographic Characteristic	Value
Mean age at test, y	26.8 ± 2.6
Gender, n	
Male	46 (46.9%)
Female	52 (53.1%)
Residency program area, n	
North	45 (45.9%)
Centre	34 (34.7%)
South	19 (19.4%)
Year of enrolment, n	
2019 (5th year)	19 (19.4%)
2020 (4th year)	34 (34.7%)
2021 (3rd year)	30 (30.7%)
2022 (2nd year)	15 (15.2%)
Sentimental relationship, n	
Single	21 (21.4%)
Stable relationship (non-marriage)	45 (45.9%)
Married	11 (11.2%)
Recent relationship	13 (13.2%)
Prefers not to answer	8 (8.3%)
Infants, n	
Yes	4 (4.1%)
No	94 (95.9%)

**Table 2:** List of survey’s main questions and related answers.

Survey Question	Answer
How would you rate the quality of theoretical education? ^†^	
Mean	5.2 ± 2.1 (median 5)
How would you rate the quality of simulation training? ^†^	
Mean	3.9 ± 2.6 (median 4)
How would you rate the quality of practical surgical training? ^†^	
Mean	5.3 ± 2.5 (median 6)
At present, how many surgical cases as first operator have you performed?	
Mean	32.5 ± 43.9 (median 20)
At present, how many surgical cases per month do you perform as first operator?	
Mean	1.97 ± 1.95 (median 1)
How many hours per month do you work?	
Mean	219.4 ± 45.3 (median 220)
Of these working hours, how many of them would you define as “administrative work”?	
Mean	85.4 ± 62.5 (median 80)
How would you rate the capacity of your School to aggregate residents? ^†^	
Mean	5.89 ± 2.7 (median 6)
How much do you feel valorized by your School?	
Mean	5.8 ± 2.4 (median 6)
Do you believe that in your educational path there was at least one person who significantly cared about your training (mentorship)?	
Yes	76 (76.8%)
No	23 (23.2%)
How would you globally rate your School and its training offer?	
Mean	6.2 ± 1.9 (median 6)
How would you rate, overall, your satisfaction for your personal life? *	
Mean	5.9 ± 1.9 (median 6)
Do you believe that your work leaves you enough free-time to enjoy your out-of-work activities? *	
Yes	21 (21.4%)
No	77 (78.6%)
How would you rate the quality of the colleagues with whom you have worked since now (both residents and surgeons)? *	
Mean	6.9 ± 1.6 (median 7)
How often has your hospital lacked a meal service for residents? *	
Never	28 (28.6%)
Rarely	12 (12.2%)
Sometimes	18 (18.4%)
Frequently	19 (19.4%)
Always	21 (21.4%)
How frequently have you considered leaving residency? *	
Never	31 (31.6%)
Rarely	26 (26.5%)
Sometimes	25 (25.5%)
Frequently	12 (12.2%)
Really often	4 (4.2%)
If you could choose again, would you choose thoracic surgery residency? *	
Yes in the same School	49 (50%)
Yes but in another School	18 (18.4%)
Yes but in another Country	14 (14.3%)
No	17 (17.3%)
How many times did your work hamper your ability to take care of your health (e.g., lost medical appointments)? *	
Never	14 (14.3%)
Rarely	24 (24.5%)
Sometimes	25 (25.5%)
Frequently	19 (19.4%)
Very often	14 (14.3%)
Prefers not to answer	2 (2%)
Have you ever received sexual harassment? *	
Yes, from physicians	4 (4.2%)
Yes, from other residents	1 (1%)
Yes, from nurses	2 (2%)
Yes, from patients	1 (1%)
No	88 (89.8%)
Prefers not to answer	2 (2%)
How commonly have you heard someone stating that thoracic surgery is not for homosexuals or women? *	
Never	45 (45.9%)
Sometimes	35 (35.7%)
Frequently	12 (12.2%)
Prefers not to answer	6 (6.2%)

^†^ These questions provided answers on a 10-point Likert scale. * These questions were used to investigate quality of life.

**Table 3:** Details of burnout risk assessment in thoracic surgery residents in Italy.

Characteristic	Value
Residents per burnout risk category, n. (%)	
High burnout risk	59 (60.2%)
Low burnout risk	39 (39.8%)
Residents with high burnout risk score per number of subscales, n. (%)	
Three subscales	6 (6.1%)
Two subscales	20 (20.4%)
One subscale	33 (33.7%)
Burnout risk score per subscale, median (interquartile range)	
Emotional Exhaustion (EE)	24.5 (IQR 16.0–31.8)
Depersonalization (DP)	38 (IQR 33.3–41.0)
Personal Accomplishment (PA)	5.5 (IQR 2.3–10.0)
Residents per Emotional Exhaustion (EE) burnout risk score, n. (%)	
High risk score	51 (52%)
Intermediate risk score	16 (16.3%)
Low risk score	31 (31.6%)
Residents per Personal Accomplishment (PA) burnout risk score, n. (%)	
High risk score	15 (15.3%)
Intermediate risk score	48 (48.9%)
Low risk score	35 (35.7%)
Residents per Depersonalization (DP) burnout risk score, n. (%)	
High risk score	31 (31.6%)
Intermediate risk score	15 (15.3%)
Low risk score	49 (50%)

**Table 4:** Multivariable analysis of variables associated with burnout risk. Model parameters represent odds ratios’ logarithms (a positive value indicates an increase in burnout risk with respect to baseline level).

Variable	Model Parameter (Standard Error)	*p*-Value for χ^2^ Test
Intercept	−3.82 (1.66)	
Administrative working hours	0.02 (0.01)	0.001
Difficulty in self-care due to workload	1.36 (0.70)	0.044
Presence of on-call and/or on-duty night shifts	1.56 (0.70)	0.019
Lack of mentorship	1.54 (0.75)	0.029
Theoretical education rating		0.060
4–5	−2.02 (0.96)	
6–7	−2.22 (0.91)	
8–10	−1.71 (1.23)	

**Table 5:** Associations between risk factors. Significant variables in both the multivariable and the univariate analyses are in the rows, while variables significant only in the univariate analysis are in the columns. The values represent the significance (*p*-value) for a χ^2^ test of independence. D = directly proportional, I = inversely proportional.

	Perception of Personal Valorization	Satisfaction for the Residency Program	Satisfaction for Personal Life	Quality of Colleagues	Dropout Contemplation	Experience of Sexual Harassment	Residency Program’s Capacity for Aggregating Residents
Theoretical education rating	<0.001 (D)	<0.001 (D)	0.006 (D)	0.015 (D)	0.217	0.081	<0.001 (D)
Night shifts	0.163	0.302	0.795	0.099	0.415	0.289	0.003 (I)
Mentorship	0.330	0.320	0.018 (D)	0.113	0.029 (I)	0.970	0.630
Difficulty in self-care due to workload	<0.001 (I)	0.446	0.341	0.082	0.595	0.829	0.010 (I)

## Data Availability

The data that support the findings of this study are available from the corresponding author, G.M., upon reasonable request.

## Supplementary Materials

The following supporting information can be downloaded at: https://www.mdpi.com/article/10.3390/healthcare13090962/s1, Supplementary Table S1. Characteristics of vacations and residents of thoracic surgery residency programs (schools of residency) in Italy at March 2024. Supplementary Table S2. Univariate analysis of variables associated with burnout risk, expressed as the proportion of individuals at risk. Supplementary Material S1. Whole survey in English, translated from Italian.

## Abbreviations

The following abbreviations are used in this manuscript:

MBI	Maslach Burnout Inventory
EE	Emotional Exhaustion
DP	Depersonalization
PA	Personal Accomplishment

## Appendix A

### Appendix A.1

The thoracic surgery residency program in Italy is a five-year, single-specialty training pathway, with admission regulated through annually held national tests. The number of available positions is limited and allocated across the country. Each university offers its own residency program, referred to as a “School of Specialization”, which must adhere to national standards established by the Ministry of University and Research [31]. Training is conducted through a network of affiliated hospitals, allowing residents to rotate through different thoracic surgery settings. Due to the single-specialty nature of the program, it may include short-term rotations in closely related specialties (e.g., cardiac or vascular surgery). The primary objective of the program is to train residents to become certified specialists in thoracic surgery. Upon successful completion of the program, residents receive a specialist qualification in thoracic surgery, which is mandatory for employment in thoracic surgery units within Italy. To be eligible for the final certification exam, residents must meet the minimum procedural requirements set by the Ministry of University and Research. These include performing, as first operator, at least 10 major surgical procedures, 30 intermediate procedures, and 80 minor procedures [31].

### Appendix A.2

SIET Residents’ Committee Collaborative Group:

Noemi Camarda: Thoracic Surgery Unit, Baggiovara Hospital, AOU Modena, University of Modena, Modena, Italy

Alessandra Cotroneo: Department of Translational Medical Sciences, University of Campania “Vanvitelli”, Monaldi Hospital, Naples, Italy

Delia Giovanniello: Thoracic surgery residency program University of Rome la Sapienza—San Camillo Forlanini Hospital, Rome, Italy

Antonio Lamesta: Thoracic Surgery Unit, Baggiovara Hospital, AOU Modena, University of Modena, Modena, Italy

Beatrice Leonardi: Department of Thoracic Surgery, University of Campania “Vanvitelli”, Naples, Italy

Fabiana Messa: Department of Thoracic Surgery, Sant’Andrea Hospital, Sapienza University, Rome, Italy

Adriana Nocera: Division of Thoracic Surgery, Fondazione Policlinico Universitario A. Gemelli, IRCCS, Rome, Italy

Letizia Perri: Thoracic Surgery Unit, Baggiovara Hospital, AOU Modena, University of Modena, Modena, Italy

Giorgia Piccioni: Department of Thoracic Surgery, Sant’Andrea Hospital, Sapienza University, Rome, Italy

Domenico Pourmolkara: Thoracic Surgery Unit, Department of Surgical Sciences, Santa Maria della Misericordia University Hospital, Perugia, Italy

Emanuele Vocale: Thoracic Surgery Unit, AOU Modena, University of Modena, Modena, Italy
