# Supplementary material for: A Nationwide Survey to Investigate Burnout and Quality of Life Among Thoracic Surgery Residents in Italy
Source: Healthcare (Basel). 2025 Apr 22;13(9):962. doi: 10.3390/healthcare13090962 (PMC12072095; doi:10.3390/healthcare13090962)
Supplement: Supplementary file 1 [file healthcare-13-00962-s001.zip › healthcare-3533269-supplementary.pdf]

Supplementary materials

Supplementary Tables

Supplementary Table S1. Characteristics of vacations and residents of thoracic surgery residency programs (Schools of residency) in Italy at March 2024.

**Year of enrolment**	**Available vacations**	**N° of Schools of residency**	**N° of present residents**	**Non-assigned vacations**	**Abandons**	**Transfers**	**Mean age (y)**
2019 (5th year)	47	16	36	2	9	1	27 ± 1.46
2020 (4th year)	80	17	64	5	11	3	27 ± 2.20
2021 (3rd year)	99	19	59	28	13	4	26 ± 3.25
2022 (2nd year)	81	19	33	46	2	0	26 ± 2.75
2023 (1st year)	93	16	?	?	?	?	?

Supplementary Table S2. Univariate analysis of variables associated with burnout risk, expressed as the proportion of individuals at risk.

Variable	Proportion at risk (standard error)	*P*-value
Gender		*P* = 0.899
Male	0.61 (0.07)	
Female	0.60 (0.07)	
Year of residency		*P* = 0.110
2nd	0.73 (0.11)	
3rd	0.70 (0.08)	
4th	0.44 (0.09)	
5th	0.63 (0.11)	
Sentimental status		*P* = 0.239
Single	0.48 (0.11)	
Longstanding relationship	0.71 (0.07)	
Married	0.55 (0.15)	
Recent/not longstanding relationship	0.62 (0.13)	
Thoracic surgery as first choice		*P* = 0.382
Yes	0.57 (0.06)	
No	0.67 (0.86)	
Residency program's geographical location		*P* = 0.295
North	0.56 (0.07)	
Centre	0.71 (0.07)	
South	0.53 (0.11)	
N° of residents per Department		*P* = 0.269
1-3	0.67 (0.08)	
4-7	0.51 (0.07)	
8-10	0.68 (0.10)	
Theoretical education rating		*P* = 0.006
0-3	0.88 (0.07)	
4-5	0.53 (0.09)	
6-7	0.49 (0.09)	

8-10	0.50 (0.16)	
Presence of simulation courses		$P = 0.297$
Yes	0.54 (0.08)	
No	0.64 (0.06)	
Practical education rating		$P = 0.216$
0-3	0.75 (0.08)	
4-5	0.62 (0.11)	
6-7	0.52 (0.10)	
8-10	0.50 (0.11)	
Cumulative procedures as first operator †		$P = 0.819$
5	0.59 (0.06)	
20	0.60 (0.05)	
50	0.61 (0.05)	
Mean monthly procedures as first operator †		$P = 0.548$
1	0.59 (0.06)	
2	0.60 (0.05)	
3	0.62 (0.06)	
Mean monthly total working hours †		$P = 0.171$
200	0.57 (0.06)	
220	0.60 (0.05)	
250	0.65 (0.06)	
Mean monthly administrative hours †		
30	0.41 (0.07)	$P < 0.001$
80	0.60 (0.05)	
140	0.80 (0.06)	
Additional working activity		$P = 0.014$
No extra activities	0.41 (0.09)	
On-call and/or on-duty night shifts	0.68 (0.06)	
Free time use for research activity		$P = 0.674$
Yes	0.61 (0.05)	

No	0.50 (0.25)	
Residency program's capacity of aggregating residents rating		*P* = 0.013
0-3	0.80 (0.09)	
4-5	0.77 (0.09)	
6-7	0.46 (0.09)	
8-10	0.46 (0.09)	
Perception of personal valorization rating		*P* = 0.001
0-3	0.89 (0.07)	
4-5	0.74 (0.09)	
6-7	0.51 (0.08)	
8-10	0.36 (0.10)	
Mentorship		*P* = 0.037
Yes	0.55 (0.06)	
No	0.78 (0.09)	
Overall residency program quality rating		*P* = 0.002
0-3	1.00 (<0.001)	
4-5	0.67 (0.09)	
6-7	0.60 (0.07)	
8-10	0.39 (0.10)	
Abroad training experience		*P* = 0.804
Yes	0.60 (0.05)	
No	0.64 (0.15)	
Overall personal life rating		*P* = 0.004
0-3	0.90 (0.09)	
4-5	0.80 (0.09)	
6-7	0.53 (0.07)	
8-10	0.40 (0.11)	
Quality of the colleagues rating		*P* = 0.023
0-3	1.00 (<0.00)	
4-5	0.83 (0.11)	

6-7	0.61 (0.07)	
8-10	0.47 (0.08)	
Absence of in-hospital restaurant service		P = 0.097
Always or often	0.53 (0.07)	
Never, rarely or sometimes	0.70 (0.07)	
Dropout contemplation		P = 0.050
Never, rarely or sometimes	0.56 (0.05)	
Really often or often	0.81 (0.10)	
Difficulty in self-care due to workload		P = 0.006
Never, rarely or sometimes	0.51 (0.06)	
Really often or often	0.79 (0.07)	
Experience of sexual harassment		P = 0.078
Yes	0.86 (0.12)	
No	0.58 (0.05)	

† For quantitative variables, the 25th, 50th and 75th percentiles were used to predict the percentage of individuals at risk of burnout.

Supplementary Materials

Supplementary Material S1. Whole survey in English, translated from Italian.

Survey for Thoracic Surgery Residents in Italy

Demographic Information

In this section, we collect information about you and your School of Residency (or residency program).

1. Enter your birth month and year. Entering the exact day is not required if you prefer not to provide it. For the month, you can choose a "near" month to your actual birth date if preferred.

2. Indicate your gender:
   - Male
   - Female
   - Other

3. Describe your sentimental status:
   - Single
   - In a long-term relationship (not married)
   - Married
   - In a recent or undefined relationship
   - Prefer not to specify

4. Do you have children?
   - Yes
   - No

5. Select the University associated with your Residency School.

6. Have you transferred to another School?
   - Yes
   - No

7. Indicate the year you took the national residency exam (SSM):
   - SSM2019
   - SSM2020
   - SSM2021
   - SSM2022
   - SSM2023

8. Was thoracic surgery your first choice of specialty?
- Yes
- No

9. Do you believe the university where you graduated provided adequate training to understand the choice of thoracic surgery?
- Yes
- No

10. Considering your entire training period (in the same or different locations), how many other residents have you worked with in the same department? Include yourself in the count.

11. Does your School have rotations within the training network (e.g., between different hospitals)?
- Yes
- No

12. If you answered "Yes", which was the mean duration of these rotations?
- < 6 months
- 6-12 months
- >12 months

Training Information

In this section we will ask you about your training experience in your School.

13. Are there lectures scheduled in your School? (Exclude sessions organized independently by hospitals or national projects like SISCT or abroad experiences.)
- Yes
- No

14. How do you rate the overall theoretical training (e.g., lectures, webinars, conferences) offered by your School? (Rate on a scale of 1 to 10; scores of 5 or below indicate inadequacy.)

15. Are practical simulation courses included in your School's training? (Exclude independently organized courses.)
- Yes
- No

16. How do you rate the simulation-based practical training offered by your School (e.g., cadaver labs, simulators, virtual reality)?

17. How do you rate your practical training as a first operator in your School?

18. At present, how many surgical cases as first operator have you performed? (Exclude chest drain placements or thoracenteses).

19. At present, how many surgical cases per month do you perform as first operator? (Exclude chest drain placements or thoracenteses).

20. On average, how many surgical procedures have you assisted with (not as the first operator) per month?

21. On average, how many hours do you work monthly (including weekends)?

22. What activities are included in your School's training? Select all that apply:
- On-duty night shifts
- On-call night shifts (e.g., transplants)
- None of the above

23. Of your monthly working hours, how many are spent on administrative tasks (e.g., scheduling tests, scanning/uploading patient documents)?

24. Of your monthly working hours, how many are dedicated to research activities (e.g., database entry, article writing, preparing presentations)?

25. Does your School provide protected time for research?
- Yes
- No

26. Have you used personal time (e.g., outside working hours) for research activities?
- Yes
- No

27. Do you believe your School encourages participation in scientific activities?
- Yes
- No

28. Has your School facilitated or at least been neutral about your attendance at conferences/seminars?
- Yes
- No, I was hindered or not supported
- Prefer not to answer

29. How do you rate the capacity of the School to aggregate and include residents (e.g., organizing events, fostering relationships among residents)?

30. Do you feel valorized by your School?

31. Do you believe that in your educational path there was at least one person who significantly cared about your training (mentorship)?
- Yes
- No

32. Does your School require passing an exam to progress to the next year?
- Yes
- No

33. Aside from exams, does your School conduct in-year assessments to evaluate

competencies? (Exclude evaluations independently organized by a specific hospital)
- Yes
- No

34. Which of the following activities does your School offer regularly? Select those where the School has developed competence (not occasional cases):
- Robotic-assisted thoracic surgery (RATS)
- Video-assisted thoracic surgery (VATS)
- Benign tracheal disease
- Malignant tracheal disease
- Benign esophageal disease
- Malignant esophageal disease
- Benign pleuropulmonary disease
- Malignant pleuropulmonary disease
- Benign mediastinal disease
- Malignant mediastinal disease
- Benign thyroid disease
- Malignant thyroid disease
- Benign chest wall disease
- Malignant chest wall disease
- Thoracic trauma surgery
- Lung transplant surgery
- Bronchoscopy (flexible and rigid)

35. In which activities do you feel your School has prepared you as an independent specialist? (For fourth- and fifth-year residents only, but a response is required to continue.)
- Clinical, ward, and outpatient activities
- Endoscopic procedures
- Minor surgeries (e.g., diagnostic thoracoscopies, atypical pulmonary resections)
- Major surgeries (e.g., lobectomies, pneumonectomies, thymectomies)
- Minimally invasive surgery
- Open surgery
- Not yet enrolled in the fourth year

36. Does your training include any legislative education relevant to your field?
- Yes
- No

37. How well do you understand your rights, duties, and national/international training goals and regulations?
- Very well
- Moderately
- Slightly
- Not at all

38. If you were to give an overall score for your School and training program, what would it be?

39. If offered a job at a hospital within your School's network, would you accept it?
- Definitely
- Likely

- Neutral
- Unlikely
- Definitely not
- Not yet enrolled in the third year

Training Abroad

In this section we will ask you about training experiences abroad.

40. Have you completed a training period abroad?
  - Yes
  - No

41. If yes, how would you compare the quality of training abroad to that provided by your School in Italy?
  - Much lower
  - Slightly lower
  - Equal
  - Slightly higher
  - Much higher

Quality of Life During Residency

42. How would you rate, overall, your satisfaction for your personal life? (Rate on a scale of 1 to 10.)

43. Do you believe that your work leaves you enough free-time to enjoy your out-of-work activities?
  - Yes
  - No

44. How would you rate the quality of the colleagues with whom you have worked with since now (both residents and surgeons). (Rate on a scale of 1 to 10.)

45. How often has your hospital lacked a meal service for residents?
  - Always
  - Frequently
  - Sometimes
  - Rarely
  - Never

46. How frequently have you taken in consideration to leave residency?
  - Very often
  - Often
  - Sometimes
  - Rarely

- Never

47. If you could choose again, would you choose thoracic surgery residency?
- Yes, at the same School
- Yes, but at a different School
- Yes, but abroad
- No

48. How many times did your work hampered to take care of your health (e.g., lost medical appointments)?
- Very often
- Often
- Sometimes
- Rarely
- Never
- Prefer not to answer

49. Have you ever received sexual harassment?
- Yes, by medical staff
- Yes, by other residents
- Yes, by nursing staff or assistants
- Yes, by patients
- No
- Prefer not to answer

50. How commonly have you heard stating that thoracic surgery is not for homosexual or women "?
- Often
- Sometimes
- Never
- Prefer not to answer

Burnout Screening

51-71. [Questions follow the Maslach Burnout Inventory format, assessing emotional exhaustion, depersonalization, and personal accomplishment scales.]

51. I feel emotionally drained from my work.
52. I feel used up at the end of the workday.
53. I feel fatigued when I get up in the morning and have to face another day on the job.
54. I can easily understand how my patients feel about things.
55. I feel I treat some patients as if they were impersonal objects.
56. Working with people all day is really a strain for me.
57. I deal very effectively with the problems of my patients.
58. I feel burned out from my work.
59. I feel I'm positively influencing other people's lives through my work.
60. I've become more callous toward people since I took this job.
61. I worry that this job is hardening me emotionally.
62. I feel full of energy.

63. I feel frustrated by my job.
64. I feel I'm working too hard on my job.
65. I don't really care what happens to some patients.
66. Working with people directly puts too much stress on me.
67. I can easily create a relaxed atmosphere with my patients.
68. I feel exhilarated after working closely with my patients.
69. I have accomplished many worthwhile things in this job.
70. I feel like I'm at the end of my rope.
71. In my work, I deal with emotional problems very calmly.
72. I feel patients blame me for some of their problems.

Possible answers:
- Never
- A few times a year or less
- Once a month or less
- A few times a month
- Once a week
- A few times a week
- Every day

This concludes the survey. Thank you for your participation!

The survey was translated from Italian to English however, the original survey in Italian language is available upon request.
